# Rapid tissue processing using a temperature-controlled collection device to preserve tumor biomarkers

**DOI:** 10.1007/s10561-019-09800-8

**Published:** 2019-12-14

**Authors:** Melissa Lerch, Heidi Kenerson, Abbey Theiss, David Chafin, Maria Westerhoff, Michael Otter, Raymond Yeung, Geoffrey Baird

**Affiliations:** 1 Department of Laboratory Medicine, University of Washington Medical Center, Seattle, WA USA; 2 Department of Surgery, University of Washington Medical Center, Seattle, WA USA; 3 Ventana Medical Systems, Inc., 1910 Innovation Parkway, Tucson, AZ USA; 4 Department of Pathology, University of Washington Medical Center, 1959 NE Pacific St., Seattle, WA USA

**Keywords:** Immunohistochemistry, Metastatic liver tumors, Hepatocellular carcinoma, Formalin fixation, Preanalytics

## Abstract

Precision tissue diagnostics rely on high quality input specimens so that assay results are not affected by artifact, but advances in collection and processing of tissue specimens have lagged behind innovations in diagnostic assay development. Therefore, we have designed and evaluated a novel surgical tissue collection device that maintains and monitors sample temperature and motion throughout transport so that the major preanalytical variable of tissue temperature can be controlled and measured. This device, in combination with an improved cold–hot tissue fixation protocol affords optimal biomarker preservation in less overall time, thereby simultaneously improving diagnostic quality and turnaround time. We collected 50 primary and metastatic liver tumors using a novel transport device. Tissue was fixed using a rapid cold–hot fixation protocol and immunohistochemical assays were used to assess the performance of the device, in comparison to control tissue preserved using standard clinical fixation protocol. Two pathologists evaluated the IHC studies in a blinded fashion to determine the immunophenotype of each tumor. The observed IHC staining intensities and the clinical impressions of the immunophenotypes did not differ between tissue collected with the novel device and control tissue, while improvements in processing time were achieved. The novel cold transport device and rapid fixation protocol can be successfully and safely combined and used to monitor specimen conditions, thus preserving the diagnostic utility of specimens and improving the overall turn-around time of the diagnostic process.

**Electronic supplementary material:**

The online version of this article (10.1007/s10561-019-09800-8) contains supplementary material, which is available to authorized users.

## Introduction

Modern cancer therapeutics target specific proteins and pathways in biochemical networks, forming the backbone of “Precision Medicine.” Effective precision medicine relies on clinical diagnostic tests to guide the choice of specific therapeutics, and such tests must be performed on high-quality tissues in which the pathophysiologic derangement responsible for the disease in question are preserved in a state that can be measured accurately (Twomey et al. 2017). One example of a precision medicine test is immunohistochemistry (IHC), such as the test for HER2-overexpression in breast carcinoma that constitutes the key piece of evidence informing a clinical decision to treat a patient with anti-HER2 therapy. HER2 assays, like all IHC assays, are susceptible to preanalytical errors such as inadequate fixation or tissue processing, yet fixation and processing steps are often de-emphasized or taken for granted as an established part of the hospital workflow (Agrawal et al. 2018). Additionally, although formalin fixation has been used for more than a century to preserve tissue for histopathology, our understanding of the biochemistry of formalin fixation is incomplete. There is evidence that cold formalin fixation improves the preservation of biochemical markers especially within signaling networks such as phosphoproteins (Chafin et al. 2013; Theiss et al. 2014), and that cold formalin fixation has been shown to aid in the preservation of nucleic acids (Bussolati et al. 2011), but ideal fixation conditions for all tissue assays have not yet been established, and the lack of current fixation monitoring technology means that even if optimal tissue fixation conditions are established for specific assays, it may be difficult to ensure that every single clinical specimens receives this optimal treatment.

Cold ischemia time, referring to the time a tissue specimen sits ex vivo prior to fixation, is another preanalytical variable that has a demonstrated and profound effect on measurements of signaling proteins like phosphoproteins (Neumeister et al. 2012; Wolf et al. 2014). There is a growing need to develop diagnostics targeting labile phosphorylated signaling proteins as more kinase inhibitors are developed into drugs, and hence there is a clinical imperative to study and develop approaches that control and monitor the temperature and time that specimens experience prior to fixation. We and others have found, for example, that rapid placement of tissues into cold formalin fixatives ameliorates some of the negative effects of prolonged cold ischemia time on measured levels of phosphoproteins, especially in larger tissue specimens that require longer fixation times (Bussolati et al. 2011; Chafin et al. 2013; Theiss et al. 2014; Gündisch et al. 2015).

We designed an approach to improving the quality of surgically-excised tissue using a cold transport device to facilitate the rapid collection, fixation, and monitoring of sensitive specimens for evaluation. We demonstrate the use of this novel cold transport device by collecting a variety of liver tumors, both primary and metastatic. We hypothesized that this device would be compatible with collection in a clinical environment, and that the tissue samples collected would provide identical clinical results as paired clinical tissue from the identical cases collected by clinical staff according to the current standard of care (including variable cold ischemic time followed by variable room temperature formalin fixation, generally overnight). The tumors in this study were generally resected for curative intent or debulking, and hence extensive diagnostic assessments were not clinically necessary. We therefore developed a contrived clinical situation in which we treated each case as a potential metastatic versus primary carcinoma, and we applied standard H&E and IHC panels to determine the carcinoma’s immunophenotype. If the histopathologic studies lead to the same clinical impression using the tissue that was collected with the clinical standard of care and the tissue that was collected with the novel collection device, then our hypothesis would be supported. The intent of this experiment was thus to test whether or not the novel collection device combined with our rapid cold–hot fixation protocol was safe and effective for providing tissue assay results that were clinically identical to the standard of care with an improved turnaround time.

## Materials and methods

### Data logger and cold storage device

**Fig. 1:**
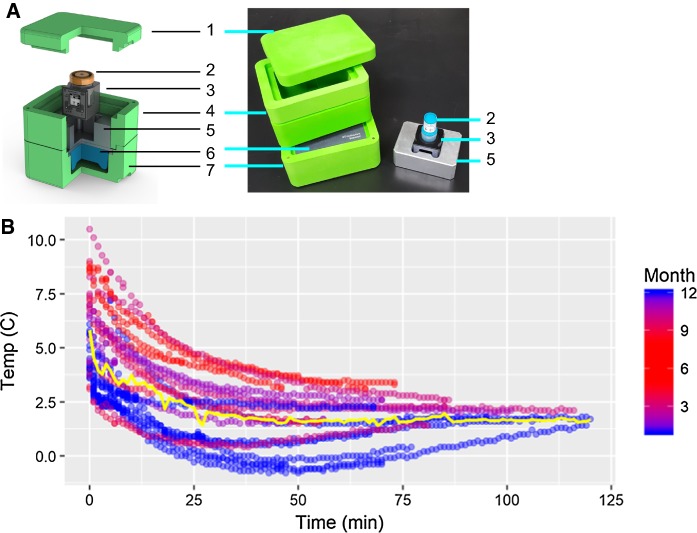
Cold transport device and data. **a** Coolbox and data logger: (1) lid (2) formalin sample container (3) datalogger (4) insulating foam box, top “Discussion” section. Aluminum datalogger holder (6) frozen cooling core (7) insulating foam box, bottom section. **b** Temperature of the data logger over time where 0 min is the sample acquisition time in the operating room. The yellow line is the average temperature of the dataset. Warmer summer months are plotted in red and cooler winter months are plotted in blue indicating a seasonal variation. (Color figure online)

The cold transport device consists of a foam-insulated box (CoolBox, Biocision) with a metal sample holder designed to fit a data logger that holds a sample collection vial (Fig. 1a). The temperature is maintained with a cooling core (pre-chilled − 20 °C) and assembled with the metal holder 20-min prior to collection. The metal sample holder, formalin, and data loggers were pre-chilled at 4 °C prior to assembly.

Data loggers have several sensors allowing collection of temperature, position, time, and other variables during transport (Fig. 1a).

### Tissue collection

**Fig. 2:**
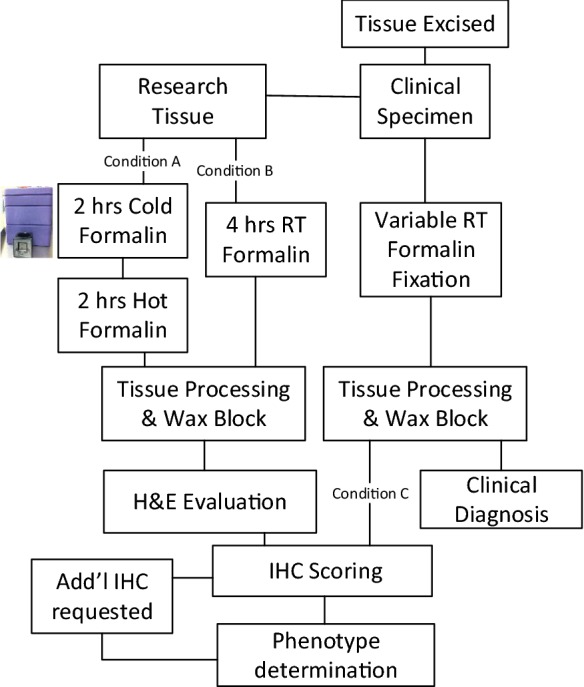
Experimental design for tissue collection, processing, and phenotype determination

Approval was granted through UWMC Institutional Review Board (#31281). Tissue was procured through the Liver Tissue Repository at the University of Washington Medical Center (UWMC) and consent was obtained from all patients prior to surgical resection to harvest fresh tissue not needed for pathologic evaluation (Fig. 2, Conditions A, B). Separate consent was obtained through UWMC tissue repository service, NW Biotrust, for the clinical specimen remaining after clinical testing (Fig. 2, Condition C).

Fresh tissue was collected directly in the operating room where a small portion of resected tissue was used for this study. The research tissue was equally divided between two conditions A and B (minimum of 4-mm core biopsies) and placed directly into dry containers with a formalin dispenser built into the lid (Biopsafe, Axlab, Denmark) to minimize formalin exposure in the operating room. After the specimen was placed in the container and closed, pressing a button on the lid punctured a receptacle containing formalin and immersed the specimen in fixative. For Condition A, the formalin was cold and the data logger was activated to record cold formalin incubation time. Tissue for Condition B was placed into room-temperature (RT) formalin. Cold ischemic time was kept to an absolute minimum by performing tissue acquisition in the operating room immediately upon tumor resection. The clinical specimen was processed as per usual clinical workflows and referred to as Condition C.

### Tissue processing

Tissue for Condition A was fixed for 2 h in 4 °C formalin and then 2 h in 45 °C formalin (2 + 2) (Chafin et al. 2013) and tissue in Condition B was fixed for 4 h at RT. Tissue was processed on a commercial tissue processor, Lynx II (Electron Microscopy Services) equipped with two Peltier stations that can cool and heat reagents. A standard overnight processing protocol was used with a variable 70% ethanol hold (10 min–6 h), 2 × 60-min 90% ethanol, 3 × 60-min 100% ethanol, 2 × 60-min xylene, 1 × 90-min xylene (45 °C) and 60-min wax. Tissue was placed into paraffin blocks and sectioned onto glass slides (4 μm). Tissue from Condition C was processed in the hospital’s clinical pathology laboratory and sectioned onto glass slides (4 μm).

### Immunohistochemistry

Immunohistochemistry was performed on an automated VENTANA Discovery XT staining instrument according to the manufacturer’s recommendations. 27 different antibodies were utilized in this study (Supplementary Table 1). Slides were deparaffinized using EZPrep (Ventana Medical Systems) at 90 °C, antigen retrieval, and antibodies conditions followed package inserts. Slides were developed using OmniMap DAB detection kit (Ventana Medical Systems) and counterstained with hematoxylin. All Conditions were stained simultaneously for all antibodies except H&E. Whole slide images were obtained using an Aperio slide scanning system.

### Slide scoring and determination of immunophenotypes

Slides were reviewed by two pathologists (MW, GB). H&E was first evaluated and a panel of IHC assays were ordered to determine the immunophenotype of the tumor, as if the case were a primary carcinoma versus carcinoma presenting as a metastasis from an unknown primary. After the initial review of the IHC slides, for which pathologists were blinded as to whether or not the tissue was from control or experimental conditions, staining intensity was scored and an immunophenotype was assigned. In some cases, an immunophenotype could not be unambiguously determined with the initial panel of tests, and additional IHC was ordered and scored. IHC stain intensity was scored using a simple semiquantitative scale (0, 1 +, 2 +, 3 +), and cases in which the two pathologists differed in assessment by more than one semiquantitative score (1 + vs. 3 +) were reviewed over a multiheaded scope and a consensus score was reached. Pathologists then determined the immunophenotype based on the IHC scores for each case and condition (Supplementary Table 2). The primary endpoint of this study was whether or not the novel collection device that employed cold, controlled fixation affected the final clinical impression of the immunophenotype compared with the impression reached by the same analyses performed on the tissue that was collected by the clinical standard of care.

## Results

### Collection of tissue

We collected tissue from 50 liver tumors over the course of 1 year from patients with liver tumors greater than 3 cm. Tissue was excluded from analysis in 10 cases (Table 1, 20%), when the patient’s tumor was not malignant (n = 3), there was no tumor present in the research tissue sample (n = 1), only one of the two tissue samples collected in the surgical suite contained carcinoma (n = 4), a post-fixation tissue processing error occurred (n = 1), the clinical tissue was not available due to incomplete consent (n = 1), and the research tissue sample was too small to meet our criteria for analysis (n = 1).

**Table 1:** Tumor phenotypes determined by pathologists

	Number of cases	Fraction of cases evaluated (%)
Gastrointestinal (GI)	16	40
Hepatic	8	20
Carcinoma	4	10
GI-pancreatic	3	7.5
Pancreatobiliary	2	5
Rare	2	5
Gynecological	2	5
Neuroendocrine	2	5
Thyroid	1	2.5
*Eliminated cases*		
No carcinoma	4	
Tumor in only one condition	4	
Clinical tissue unavailable	1	
Too small	1	

### Cold storage and transport

Tissue was collected directly in the operating room by placing resected material into either cold formalin (Condition A) or room temperature formalin (Condition B, Fig. 2). Condition A tissue was maintained at the same temperature by transporting within the cold transport device with a custom data logger that records the time of fixation, temperature, and transport specific parameters (including leaked fixative or aberrant acceleration, i.e. “dropping” the specimen). Condition B tissue was fixed for 4 h at room temperature and thereafter processed the same as the cold formalin sample.

Temperature profiles were obtained for tissue collections in the operating room (Fig. 1b). The temperature of the Coolbox ranged from 10 to 2.5 °C when the sample was loaded into the logger and dropped to a low temperature ranging between 3 and − 0.5 °C after 30 min. A seasonal temperature effect was observed, with specimens reaching slightly colder temperatures in colder months. All temperature profiles plateaued around 2.5 °C after 70 min in the Coolbox collection device. Most tissue samples were loaded onto the tissue processor to complete the cold fixation step before 2-h incubation was complete, meaning that the cold formalin fixation step was completed on the tissue processor followed by a 2 h fixation in hot formalin (45 °C).

### Pathologist slide scoring and phenotype determination

Two pathologists scored the slides independently using a semiquantitative scale with the intent of determining an immunophenotype while comparing the quality of slides staining for each condition. Pathologists were provided with three tissue specimens for each case: Condition A (2 + 2 cold/hot fixation), Condition B (4 h RT fixation), and Condition C (clinical specimen). Pathologists were blinded to tissue treatment condition. Twenty-seven different stains were used in this study to determine immunophenotypes, although each IHC antibody was not applied to each case (Supplementary Table 1). Five antibodies accounted for 73% of the slides. The antibody panel including CK20, CK7, CDX2, TTF1, and ER were primarily used for the phenotypic determination. The majority of intensity scores were exact matches between pathologists, accounting for 80% of slides (513 slides). Consistency increased to 90% when scores were deemed a match if they were within one semiquantitative score of each other (578 slides). The remaining 60 slides (10%) were two or more points different, which on reconciliation over a multiheaded scope were found to represent different impressions of the overall intensity of staining when only rare or focal staining was present in a small portion of a tumor. Comparisons of the scoring between the two experimental conditions resulted in differences in 10 cases for one pathologist and 12 cases for the other pathologist but the majority were due to tumor heterogeneity where a biomarker was present in only one condition rather than a difference in staining quality. Overall, there were no significant diagnostic discrepancies between pathologists resulting from IHC staining quality.

**Fig. 3:**
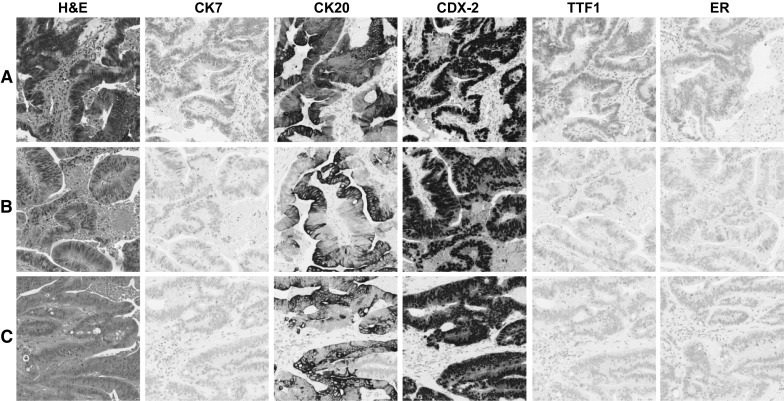
Typical gastrointestinal phenotype. Condition **A** (***rowA***) 2 h cold formalin followed by 2 h hot formalin. Condition **B** (***rowB***) 4 h room temperature formalin. Condition **C** (*rowC*) Clinical control sample. **IHCstains** H&E, CK7, CK20, CDX-2, TTF1, and ER

**Fig. 4:**
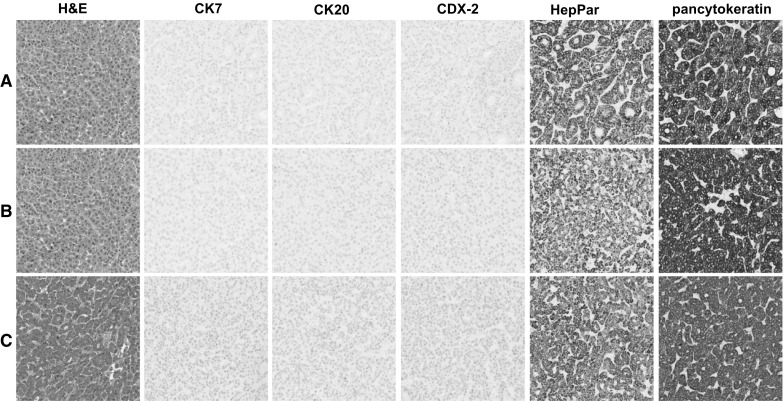
Typical hepatocellular phenotype. Condition **A** (***rowA***) 2 h cold formalin followed by 2 h hot formalin. Condition **B** (***rowB***) 4 h room temperature formalin. Condition **C** (*rowC*) Clinical control sample. **IHCstains** H&E, CK7, CK20, CDX-2, HepPar, and pancytokeratin

**Fig. 5:**
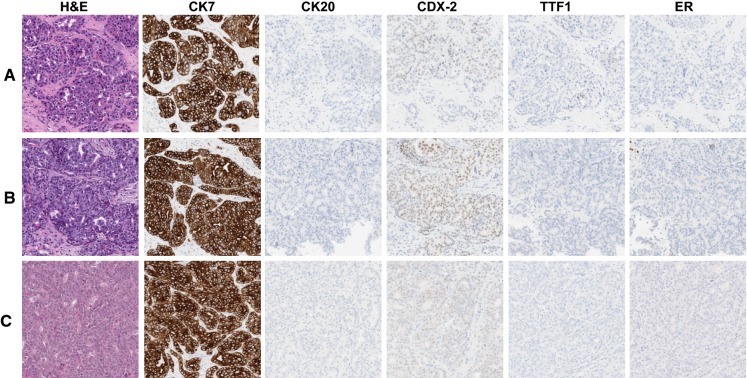
Pancreatobiliary-gastrointestinal phenotype. Condition **A** (***rowA***) 2 h cold formalin followed by 2 h hot formalin. Condition **B** (***rowB***) 4 h room temperature formalin. Condition **C** (*rowC*) Clinical control sample. **IHCstains** H&E, CK7, CK20, CDX-2, TTF1, and ER

The majority of cases collected in this study were gastrointestinal metastatic lesions in the liver (n = 16, 40%, GI, Table 1) where the typical colorectal immunophenotype of strong positive IHC staining for CK20 and CDX-2 (Fig. 3) was observed. Hepatocellular carcinoma cases were second most common (n = 8, 20%) where the typical hepatocellular immunophenotype of strong positive IHC staining for HepPar and pancytokeratin (Fig. 4) was observed. The remaining cases were determined to have pancreatobiliary (n = 2, 5%), rare/unusual (n = 2, 5%), non-specific (n = 4, 10%), gynecological (n = 2, 5%), neuroendocrine (n = 2, 5%), and thyroid (n = 1, 2.5%) immunophenotypes. There were three cases that could not be specifically categorized further than gastrointestinal-pancreatobilary with expression of CDX-2, either CK7 or CK20 or both CK7 and CK20 (Fig. 5). The non-specific carcinoma immunophenotype only showed expression of CK7 (Supplementary Fig. 1). In all cases, the immunophenotypes determined for each case was identical across tissue fixation treatments, the novel collection device produced tissue that yielded the same clinical impression as tissue collected during standard clinical operations.

## Discussion

In this study we have demonstrated the use of a cold collection transport device coupled with a rapid cold–hot formalin fixation and a standard processing protocol to generate high-quality tissue specimens for rapid turn-around of sensitive clinical diagnostic results. We found that the novel device, paired with our previously published and validated fixation conditions, resulted in high quality tissue that was clinically equivalent to tissue collected by the current clinical standard of care.

This study also focused on larger tumors that contained ample tissue for diagnostic and research specimens, with a minimum thickness of 4 mm to simulate the larger specimens that typically result from a large surgical tumor resection. Larger tissue specimens require longer fixation to allow formalin to diffuse completely into the tissue. Cold formalin fixation can mitigate some of the ischemic time affects by allowing tissue penetration while the temperature is low and degradation processes are slowed (Chafin et al. 2013).

We used a panel of standard IHC markers to distinguish primary hepatocellular carcinoma from other common and unusual metastatic carcinomas to the liver, and our novel collection device and rapid fixation strategy did not compromise clinical value of the collected tissue in any measurable way. It is known that the majority of IHC assays used for immunophenotypic determinations in the clinic are relatively robust, even though standardization of procedures across institutions could improve quality (Lin and Chen 2014). Many signaling proteins and biomarkers are labile, however, and require specific and stringent preanalytical conditions in order to be useful as diagnostics. We did not assess known labile biomarkers, such as phosphoproteins, in this study since these are not part of any current clinical histopathologic assessments, and thus comparing performance of such assays would not demonstrate the safety or effectiveness of the new device in supporting current clinical workflows. Our prior work^4^, however, has demonstrated enhanced phosphoprotein preservation resulting from the cold–hot fixation protocol, and thus we believe that with this foundational study demonstrating first the safety and compatibility of the collection device with current surgical and histopathologic workflows, we are now justified in undertaking a more comprehensive study of labile tumor biomarkers in a less contrived study, such as a clinical trial that relies on tissue collected and immediately preserved in the operating room for later phosphoprotein IHC analysis to inform a therapeutic decision.

The cold transport device aims to provide capability, performance, reliability, and flexibility to accommodate the various needs in different clinical and research environments. The transport box has a small footprint to accommodate the limited space available in operating rooms and can be sterilized. The unique formalin dispenser minimizes exposure by dispensing formalin only after the specimen is inside the container. Compared with conventional formalin containers found in operating rooms, the cold transport device was equally user friendly and compact. Its simplicity was easily adopted by the operating room staff. By initiating fixation in the operating room, we were able to complete tissue processing within 17 h of surgical excision from the patient. In eliminating a 24-h tissue fixation step, we effectively reduced the turnaround time of the clinical diagnostic workflow by 1 day while maintaining excellent tissue quality and tumor biomarker preservation (Patel et al. 2012). In a related project, we are developing an instrument for real-time monitoring of tissue fixation and processing (Lerch et al. 2016). By combining these technologies, we aim to produce high-quality tissue that is monitored at all stages in the hospital post-excision, while reducing the overall turn-around time of disease diagnosis adding a critical component to the personalized medicine toolbox.

Degradation of biomarkers during warm and cold ischemic times may confound evaluation of excision tissues. Signaling proteins are more susceptible to the effects of ischemic time (Neumeister et al. 2012). Our approach eliminated cold ischemic time associated with routine pathological evaluation by facilitating controlled and monitored collection directly in the operating room, thus beginning fixation rapidly to reduce excessive biomarker degradation.

According to a study monitoring errors in a clinical pathology laboratory, the majority of errors were identified in specimen labeling, collection and preservation and transport (Steelman et al. 2016), and thus this device, which allows for monitoring tissue temperature and time of fixation is directly responsive to the needs of the clinical laboratory. Appropriate monitoring of specimens during transport should reduce errors and lead to better patient care. The cold transport device could also be a useful tool to add to the clinical pathology laboratory toolbox to standardize processes and reduce the opportunities for deviations from processing protocols. Preanalytical monitoring allows quality assurance measures in the clinical laboratory and enables laboratories to demonstrate and document regulatory compliance, as well as enable biorepositories to curate their collections of biospecimens stored for research.

In the future, we believe that the use of a comprehensive biospecimen workflow, starting with preanalytical innovations such as temperature-controlled and monitored collections devices, optimized and rapid cold–hot fixation, and real-time fixation monitoring, will be useful in a growing number of applications. Areas that could be improved with this workflow include studies of tumor heterogeneity or biopsy-resection discordance, where it is difficult to understand currently if observed heterogeneity or discordance is pathophysiologic or artifactual, and also studies that rely on identification or quantification of labile biomarkers to inform treatment decisions.

## Electronic supplementary material

Below is the link to the electronic supplementary material.

**Supplementary Figure 1Typical non-specific carcinoma phenotype** Condition **A** (***row A***) 2 hours cold formalin followed by 2 hours hot formalin. Condition **B** (***row B***) 4 hours room temperature formalin. Condition **C** (*row C*) Clinical control sample.**IHC stains** H&E, CK7, CK20, CDX-2, TTF1, and ER. (EPS 6489 kb)

SupTable 1: Antibodies used in this study. SupTable 2: Detailed list of phenotypes comparing pathologists scoring to the pathology report (XLSX 19 kb)

## Abbreviations

UWMC: University of Washington Medical Center
IHC: Immunohistochemistry
H&E: Hematoxylin and eosin
FFPE: Formalin fixed paraffin embedded tissue
HER2: Human epidermal growth factor receptor 2
ER: Estrogen receptor
CK7: Cytokeratin 7
CK20: Cytokeratin 20
CDX2: Caudal type homeobox 2
TTF1: Transcription termination factor 1
GI: Gastrointestinal
